# Structural and functional characterisation of isolated puff adder (*Bitis arietans*) venom serine proteases

**DOI:** 10.1016/j.toxcx.2026.100263

**Published:** 2026-09-01

**Authors:** Mark C. Wilkinson, David T.K. Waterhouse, Cassandra M. Modahl, Anthony J. Saviola, Frank-Leonel Tianyi, Robert A. Harrison, Nicholas R. Casewell

**Affiliations:** a Centre for Snakebite Research and Interventions, Liverpool School of Tropical Medicine, Liverpool, UK; b Deptartment of Tropical Disease Biology, Liverpool School of Tropical Medicine, Liverpool, UK; c Department of Biochemistry and Molecular Genetics, University of Colorado Denver, 12801 East 17th Avenue, Aurora, CO, 80045, USA

**Keywords:** *Bitis arietans*, Serine protease, Gelatinase, Snake venom, Kallikrein-like

## Abstract

Serine proteases (SVSPs) are known to play a major role in the haemotoxic actions of viper venom, but compared with those from other medically important vipers, the serine proteases of puff adder venoms have not been extensively characterised. To address this, we isolated, identified and characterised the bioactivity of the serine proteases within the venom of the Nigerian puff adder, which we had previously shown to be especially rich in this class of toxin. Two distinct groups were identified, each with different protein substrate specificities. Both had similar molecular weights of 52-62 kDa, with 4-6 N-glycans, but one group consisted of trypsin-like acidic SVSPs and the other of non-trypsin-like basic SVSPs with a specificity for aliphatic amino acids at the P_1_ position. Each acted differently on fibrinogen: the acidic SVSPs showed thrombin-like alpha/beta-fibrinogenase activity, whereas the basic forms were shown to be alpha-fibrinogenases. The acidic SVSPs acted on a chromogenic substrate commonly used to determine kallikrein-like activity but also possess strong gelatinase activity - a novel activity for SVSPs and the first example of an SVSP acting on proteins other than those of the haemostatic system. The basic SVSPs were able to specifically convert angiotensin I into the vasoconstrictor angiotensin 1-8. Analysis of the transcripts of each set of SVSPs revealed structural details of the substrate-binding sites that supported the experimental findings. The activity and sequences of the basic SVSPs show that they are very like the alpha-fibrinogenase ML-AF of *M. lebetina*, which until now was considered to be a unique SVSP. Thus, this basic SVSP and the acidic SVSP with its gelatinase activity can be considered to be atypical viper serine proteases. The levels of these SVSPs in venom were found to vary geographically and this, alongside the regional variation in the SVMP activities that we observed in a previous study, is discussed with reference to the potential implications on pathology of snakebite envenoming and the development of therapeutic interventions.

## Highlights

• Puff adder venom possesses distinct and atypical acidic and basic SVSPs.
• The acidic, kallikrein-like form is a gelatinase: a novel activity for SVSPs.
• The basic form is very like the unique *M. lebetina* alpha-fibrinogenase ML-AF.
• Biochemical assays suggest that they are likely to play a role in haemotoxicity

## Graphical abstract

**Figure:**
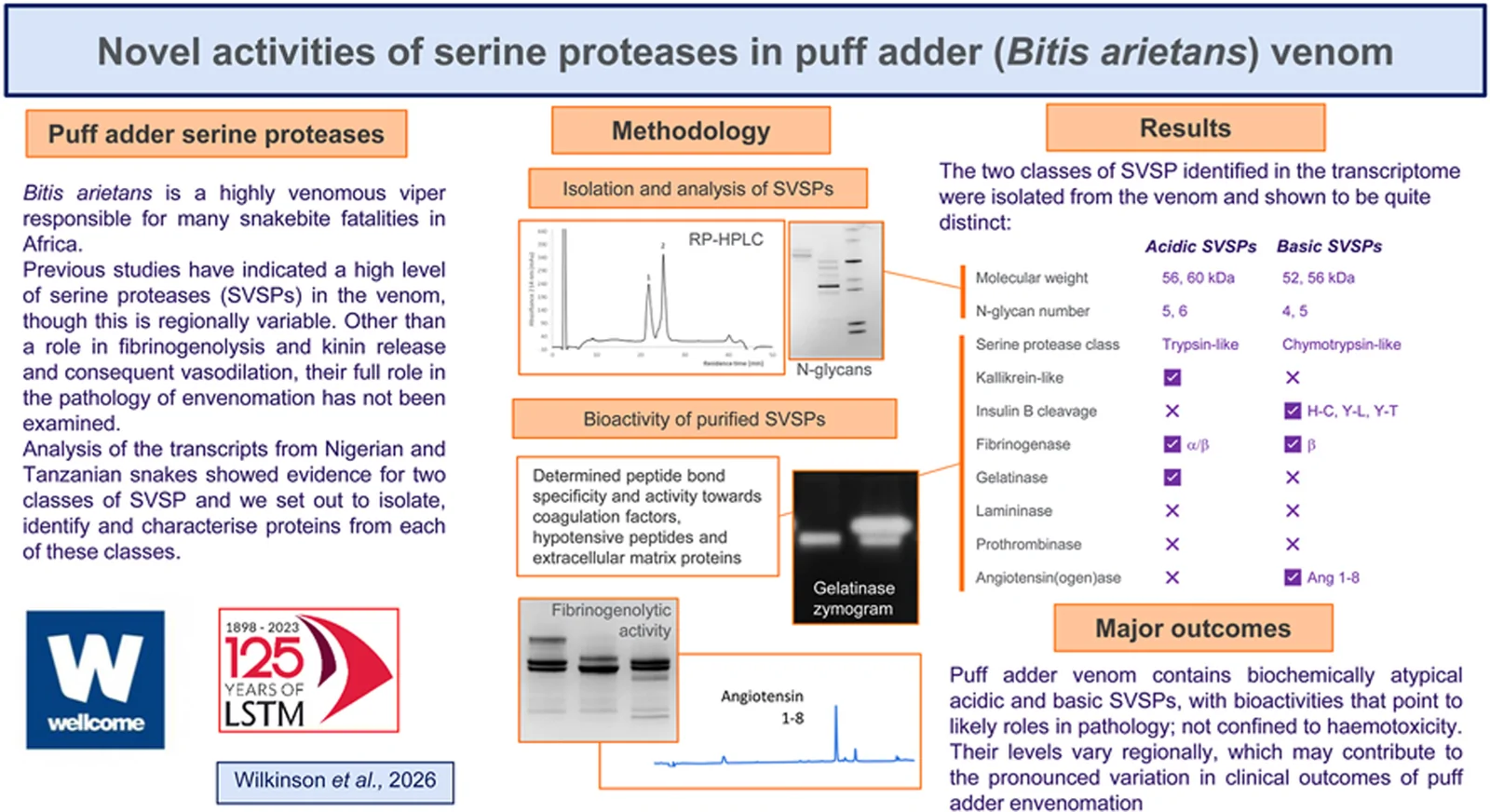

## 1 Introduction

Snake venom serine proteases (SVSPs) are found ubiquitously in the venoms of the terrestrial snake families but are especially abundant in the Viperidae (see Serrano and Maroun, 2005; Kini, 2005, for reviews). All viper SVSPs are based on a simple structural layout: a ∼26 kDa monomeric protein segment with a variable N-glycan content that results in a large range of molecular weights, from 28 to 67 kDa. From this basic structure arises a surprising variety of functions, however, all of which can disrupt one or more of the components of haemostasis. Thus, isolated viper SVSPs have been shown to have fibrinogenase activity (alpha-, beta- or less commonly alpha/beta-), fibrinolytic (via plasmin activation), platelet-activating (via PAR activation) or kallikrein-like activities (Serrano and Maroun, 2005; Kini, 2005). Most viper SVSPs are trypsin-like in their action and it is not surprising, therefore, that they have also been found to activate some of the coagulation cascade clotting factors, including prothrombin and factors X (Marrakchi et al., 1995), V (Kisiel, 1979; Siigur et al., 1998) and VIII (Niewiarowski et al., 1979; Hill-Eubanks et al., 1979). There have been a few reports of viper SVSPs that are non-trypsin-like in their substrate specificity, but these also have also been shown to act on plasma proteins such as fibrinogen (see Discussion).

The SVSP content of venom from the large *Bitis* spp. varies from ∼20 to 26%, and in *B. gabonica gabonica* it is the major venom component, exceeding that of the SVMP content (Calvete et al., 2007). The only study that has made a direct estimate of the SVSP content of puff adders (*B. arietans*) is that of quoting 19.5% for Ghanaian snakes, less than that of the SVMP and CLP content. A study on a Nigerian venom using the same methodology (Casewell et al., 2014, Supplement) indicates a similar level in these puff adders. The SVSP content of puff adders is clearly geographically variable, however. Thus, in a recent study which revealed substantial intraspecific variation in toxin expression in African puff adders, the level of both SVSP transcript expression and trypsin-like SVSP activity was ∼5-fold greater in Nigerian snakes than in those from Tanzania (Dawson et al., 2024). The complex nature of this Nigerian *B. arietans* SVSP component has been highlighted by proteomic analysis (Fasoli et al., 2010). This study used 2D PAGE for a high-resolution separation of the venom proteins for identification via mass spectrometric analysis and identified multiple SVSP proteo- and glycoforms, grouped into 5-6 glycosylation trains with an overall range of isoelectric points between 4 and 7.5, and molecular weights of 37 to 60 kDa.

Previous studies using traditional chromatographic methods to isolate and characterise individual puff adder SVSPs have shown that, in addition to fibrinogenolytic activity, they also possess kallikrein-like (kinin-releasing) activity (Sekoguchi et al., 1986; Nikai et al., 1993 Megale et al., 2018). Such SVSPs were isolated and found to possess a wide range of molecular weights, from 33 to 58 kDa, due to differences in the degree of N-glycosylation, as highlighted above. Considering the established functional diversity of SVSPs and the demonstrated complexity and content of the puff adder SVSPs, it is unlikely that fibrinogenolytic and kinin-releasing activity is their only role in puff adder venom, however. All the aforementioned studies have provided some insight into the role of SVSPs in the toxicity of puff adder venom but a complete picture of the SVSPs and their precise bioactivities is required to fully understand the diverse potential pathological effects of the venom and identify potential therapeutic targets. Here, we describe the purification of, and the structural and functional characterisation of puff adder SVSPs. The study was prompted by our observation of pronounced differences in PMSF-inhibited gelatinase activities of venom from Nigerian and Tanzanian snakes (Wilkinson et al., 2026). The bulk of the work described here focusses on the serine proteases in venoms from the Nigerian snakes, since a directly related transcriptome was available that enabled a full analysis of the variety of SVSPs and their functions.

## 2 Materials and methods

### 2.1 Venom

All venoms were collected either prior to or in accordance with the Nagoya protocol. Venoms were extracted from five Nigerian (NGA) and four Tanzanian (TZA) *B. arietans* specimens maintained within the herpetarium at the Liverpool School of Tropical Medicine (LSTM). Venoms from individual specimens were labelled, immediately frozen at −20°C and later lyophilised for long term storage at 2 – 8°C. To deliver the volume of venom required for the multiple analyses described below, venom extractions were performed on multiple occasions from each snake. The other venoms used in this study (for the gelatin zymograms, Fig. S1) were taken from stocks held at LSTM; these are either from snakes previously kept in the herpetarium (Ghanaian and Nigerian) or samples taken over many years from wild-caught animals and supplied to LSTM.

### 2.2 Reagents

All reagents used were of analytical reagent grade and, unless otherwise specified, were purchased from Merck Life Science, Watford, UK or Fisher Scientific, Loughborough, UK.

### 2.3 Serine protease purification

All chromatography was carried out using either an ÄKTA LC system (Cytiva) or a Vanquish HPLC system (Thermo Fisher). Chromatography buffers were freshly prepared and vacuum filtered (0.1 μm) immediately prior to use. The initial step in the isolation procedure was a separation of whole venom on a size exclusion chromatography (SEC) column. Twenty mg of freeze-dried venom was resuspended in 1.5 mL ice-cold 50 mM sodium phosphate pH 5.2 and centrifuged at 10,000 xg for 10 min. The supernatant was immediately loaded onto a 120 mL column of Superdex 200HR equilibrated in 50 mM sodium phosphate pH 5.2. The column was operated at a flow rate of 1.0 mL/min and 2 mL fractions were collected after the void volume. Elution was monitored at 214 and 280 nm. SDS-PAGE analysis and protease assays were carried out on all protein-containing fractions to determine which to select for the second stage of isolation. For all venoms, serine protease (SVSP) activity, indicated by PMSF-inhibited casein degradation, was found in the first main peak of the SEC separation, with bands visible on SDS-PAGE in the 50-60 kDa range (reducing conditions). These were pooled and applied to a 1 mL HiRes Capto S column equilibrated in 50 mM sodium phosphate pH 5.2. The unbound material in the flow-through (acidic SVSPs) was retained and then elution of the bound (basic) SVSPs was carried out using a 25-column volume (CV) gradient of 0 - 0.70 M NaCl in 50 mM sodium phosphate pH 5.2. The flow rate was 0.6 mL/min. The acidic SVSPs in the unbound fraction were immediately buffer exchanged into 50 mM Tris-Cl, pH 8.5 using a 50 mL Sephadex G25 desalting column. These were then loaded onto a 1 mL Mono Q column equilibrated in the same buffer. The acidic SVSPs were eluted from the column using a 25 CV gradient of 0-0.4 M NaCl in 50 mM Tris-Cl, pH 8.5. The column was operated at 0.6 mL/min and 0.5 mL fractions were collected. Elution was monitored at 214 and 280 nm. The final purification step on reverse-phase HPLC (RP-HPLC) was performed using a Biobasic C4 column (2.1 × 150 mm, Thermo Fisher). The flow rate was 0.2 mL/min and proteins were separated in the following gradient of acetonitrile in 0.1% trifluoroacetic acid; 0-35%/5 min; 35-44%/30 min; 44-72%/2 min. Elution was monitored at 214 nm.

### 2.4 Analytical methods

#### 2.4.1 SDS-PAGE

Venoms were freshly reconstituted in PBS (50 mM sodium phosphate, 0.15 M NaCl pH 7.2) and maintained on ice. Samples were prepared for reducing SDS-PAGE by adding sample buffer to a final concentration of 2% SDS, 5% β-mercaptoethanol and then heating at 85°C for 5 min. For non-reducing gels the heat treatment was omitted. Electrophoresis was performed on 4-20% acrylamide gels (BioRad TGX) using a Tris-glycine buffer system, followed by staining with Coomassie Blue R250. The molecular weight markers used were either Thermo-Fisher PageRuler or Promega Broad Range.

#### 2.4.2 Deglycosylation of isolated proteins

In preparation for deglycosylation, a 20 μL sample was denatured by heating for 5 min at 85°C following the addition of 2.0 μL 1% SDS and 0.5 μL 1 M DTT. After cooling to room temperature, 1.7 μL of 10% NP-40 was added and then 0.5 μL PNGase F (at the supplied concentration) was added to start the reaction. Incubation was carried out at 42°C for 3 h or overnight at room temperature. Where deglycosylation was carried out under native conditions for gelatin zymograms (Fig. 8), the denaturing step was omitted and deglycosylation was performed at 37°C.

#### 2.4.3 Trypsin digestion and MS/MS analysis

In preparation for this, the relevant proteins were desalted on a RP-HPLC column, as above. These were then dried in a centrifugal evaporator, 20 μL H_2_O was added and then re-dried. The proteins were then resuspended in 8 M urea/0.1 M Tris-Cl (pH 8.5), reduced with 5 mM TCEP (Tris (2-carboxyethyl) phosphine) for 20 min and alkylated with 50 mM 2-chloroacetamide for 15 min in the dark at room temperature. Samples were diluted 4-fold with 100 mM Tris-Cl (pH 8.5) and digested with trypsin at an enzyme/substrate ratio of 1:20 overnight at 37°C. The reaction was terminated by addition of formic acid (FA, final 0.5%), and digested peptides were loaded on to Evotips and analysed directly using an Evosep One LC system (Evosep Biosystems, Denmark) coupled with timsTOF SCP-mass spectrometer (Bruker, Germany). Peptides were separated on a 75 μm i.d. × 15 cm column packed with 1.9 μm C18 beads (Evosep Biosystems, Denmark) and over a 44-min gradient. Buffer A was 0.1% FA in water and buffer B was 0.1% FA in acetonitrile. Instrument control and data acquisition were performed using Compass Hystar (version 6.0) with the timsTOF SCP operating in data-dependent acquisition mode.

Fragmentation spectra were searched against an in-house *B. arietans* venom gland derived protein sequence database sourced from Nigerian specimens (see Dawson et al., 2024) using Mascot (Perkins et al., 1999). Reverse decoys and contaminants were included in the search database. Cysteine carbamidomethylation was selected as a fixed modification, oxidation of methionine was selected as a variable modification. The precursor-ion mass tolerance and fragment-ion mass tolerance were set at 10 ppm and 0.04 Da, respectively, and up to 2 missed tryptic cleavages were allowed. Mascot files were parsed into Scaffold (version 5.0.1, Proteome Software, Inc.) for validation at a protein-level false discovery rate (FDR) of < 1%. The mass spectrometry data have been deposited to the ProteomeXchange Consortium via the PRIDE (Perez-Riverol et al., 2025) partner repository with the dataset identifier PXD083303.

#### 2.4.4 General protease assays

To distinguish serine protease activities from those of the metalloprotease, the ability of the purified proteases to degrade casein and insulin B was determined in the presence of EDTA (metalloprotease inhibitor) or PMSF (serine protease inhibitor). Insulin B degradation was also used to give an indication of the degree of specificity of action of the protease. Digestion of angiotensin I was tested because of the well-documented hypotensive action of puff adder venoms, but also to provide further information on peptide bond specificities. Chromogenic substrates, each with a different amino acid in the key position, were used to determine the substrate specificity of the SVSPs allowing their classification as trypsin-, chymotrypsin- or elastase-like, and to determine potential kallikrein-like activity.

##### 2.4.4.1 Casein degradation with SDS-PAGE analysis

Venom or protein samples were incubated in TBSC (50 mM Tris-Cl, 0.15 M NaCl, 1 mM CaCl_2_, pH 7.4) with beta casein at a ratio of 30:1 (w/w) casein:sample protein; using a final concentration of 1.0 mg/mL casein. Incubation was carried out at 37°C for 2 h. SDS-PAGE sample buffer was added (final concentration 2% SDS, 5% β-mercaptoethanol) to stop the reaction. This was then heated at 85°C for 5 min. The extent of casein degradation was assessed using SDS-PAGE (see 2.4.1). In EDTA inhibition experiments, EDTA was added to the protein sample to a final concentration of 5 mM and incubated for 15 min prior to the addition of casein to start the reaction. Where PMSF was used as an inhibitor, this was added to a final concentration of 2 mM from a freshly prepared 20 mM stock solution in methanol. This pre-incubation was carried out for 60 min (room temperature) prior to addition of casein.

##### 2.4.4.2 Insulin B, angiotensinogen or angiotensin I digestion with RP-HPLC-UV and RP-HPLC-MS analysis

Venom or protein samples were incubated in TBSC with the peptides insulin B chain, angiotensinogen N-terminal tetradecapeptide 1-14 (porcine sequence DRVYIHPFHLLVYS) or angiotensin I (1-10, DRVYIHPFHL) at a ratio of 30:1 (w/w) peptide:sample protein, using a final concentration of 0.02 mg/mL peptide substrate. Where inhibitors were used (insulin B assay only), these were pre-incubated with the protein sample as in section 2.4.4.1. Incubation was carried out at 37°C for 90 min and the reaction was stopped by the addition of trifluoracetic acid (TFA) to 1%. An aliquot containing the equivalent of 1 μg insulin B/angiotensin I was analysed by RP-HPLC-UV using a Biobasic C4 column (2.1 × 150 mm). The flow rate was 0.3 mL/min and the separation was carried out with the following gradient of acetonitrile in 0.1% trifluoroacetic acid; 0-36%/40 min; 36-70%/3 min. Elution was monitored at 214 and 280 nm. RP-HPLC-MS experiments were performed on a Thermo Vanquish HPLC system (Thermo, Germering, Germany) coupled to an Orbitrap Exploris 240 mass spectrometer (Thermo, Bremen, Germany). RP-HPLC separation was carried out on a Hypersil Gold C18 column (175 Å pore size, 100 × 2.1 mm, 1.9 μm particle size) with the column oven set to 50°C. The flow rate was 0.3 mL/min and the proteins/peptides were eluted with the following gradient of acetonitrile in 0.1% formic acid: 2–40%/10 min, 40–80%/2 min, 80-0%/2 min. ESI settings were as follows: sheath gas, 5 arbitrary units (Arb); auxiliary gas, 2 Arb; spray voltage, 3.5 kV; ion transfer tube temperature, 275°C; mass tolerance, 15 ppm. The Orbitrap full scan acquisition parameters were: resolution, 120,000 (at m/z 200); scan range, m/z 200–2000; radio frequency lens (%) set to 70, polarity positive with the automatic gain control (AGC) target set to standard.

##### 2.4.4.3 Chromogenic substrate assays

Assays using the chromogenic substrates BAEE (N-alpha-benzoyl-L-arginine ethyl ester) and BTEE (N-benzoyl-L-tyrosine ethyl ester) were carried out in TBSC with the substrate at a concentration of 0.25 mM and the protease at a final concentration of 2-5 μg/mL. The reaction was followed in quartz cuvettes at 253 nm (BAEE) or 256 nm (BTEE). Where the general nitroanilide esters (N-succinyl-A-A-A-p-NA, N-methoxysuccinyl-A-A-P-V p-NA, N-succinyl-A-A-P-L p-NA, N-succinyl-A-A-P-F p-NA) were used the final concentrations were 0.2 mM substrate in TBSC and 2-5 μg/mL protease. The plasma protease substrates (Chromogenix) S-2288, HCl-I-P-R-p-NA (general serine protease substrate) and S-2302, HCl-P-F-R-pNA (for kallikrein-like activity) were used at final concentrations of 2 mM substrate and 2-4 μg/mL protease. The reaction for the nitroanilide esters was followed at 410 nm. In all cases specific activity was calculated as μmol product formed per minute per mg protease. The mM extinction coefficients used for this calculation were BAEE, 1.070 (253 nm); BTEE, 0.964 (256 nm) and for all the nitroanilides, 8.800 (410 nm). The standard serine proteases used for this section of the work were: porcine trypsin (Sequencing grade, Promega UK), TLCK-treated bovine chymotrypsin and porcine elastase.

#### 2.4.5 Functional protease assays

Four assays were carried out to determine the abilities of the SVSPs to degrade proteins that may be of functional and pathological significance. Degradation of gelatin (predominantly collagen I) or laminin-rich basement membrane material are possible indicators of the ability to cause tissue damage. Potential coagulopathic activities were measured by the ability to degrade the key coagulation factors, fibrinogen and prothrombin. In the latter case, the specific proteolytic action required to generate thrombin can be determined.

##### 2.4.5.1 Gelatin zymogram assay

The gels (10% acrylamide) for this assay were prepared using the method of Laemmli (1970). Gelatin was prepared by briefly heating a 20 mg/mL solution at 50°C, then adding this to the gel mix immediately prior to casting, such that the final gelatin concentration was 2 mg/mL (0.2% w/w). Where inhibitors were tested, these were pre-incubated with the protein sample as in section 2.4.4.1. Venom or protein samples were then prepared for the zymogram assay by adding standard SDS-PAGE sample buffer, but with no reductant and a final SDS concentration of 1.6% (w/v). These were not heated. Marker proteins were run under reducing conditions at the right hand end of the gel and no samples were run in the lane adjacent to the marker lane. Following electrophoresis, the method of Toth et al. (2012) was used to visualise gelatinase activity. Thus, the gel was rinsed, with shaking, in 2.5% (v/v) Triton X-100 for 30 min, then washed (3 × 10 min) with distilled water to remove the detergent. The buffer used for development was 50 mM Tris-Cl, 200 mM NaCl, 5 mM CaCl_2_, 0.02% (v/v) Brij 100, pH 7.8. The gel was washed for 10 min in this buffer, then this was replaced with fresh development buffer and incubated overnight at 37°C. To visualise gelatin degradation zones, the gel was stained for 60 min with Coomassie Blue R250 and destained until clear bands were visible against a blue background.

##### 2.4.5.2 Digestion of basement membrane proteins

Basement membrane material (Geltrex, Thermo Fisher) was diluted with PBS to 10% of its supplied concentration (10-18 mg/mL) and stored in aliquots at −20°C. For the assay, a fresh aliquot was carefully thawed out and immediately placed on ice. Digestions were set up with basement membrane material:protein sample at 30:1 (w/w) and performed at 37°C for various time periods between 2 min and 4 h. Following this, SDS-PAGE sample buffer was added and the sample heated at 85°C to stop the reaction. The extent of basement membrane protein degradation was then assessed using SDS-PAGE (see section 2.4.1).

##### 2.4.5.3 Digestion of plasma proteins (prothrombin and fibrinogen)

In both the prothrombin and fibrinogen degradation assays, these two substrate proteins were used in the assay at a final concentration of 1.0 mg/mL in TBSC and venom/pure protein samples were added at a ratio of 30:1 substrate:protein sample to start the reaction. Incubation was carried out at 37°C for 60 min. Following this, SDS-PAGE sample buffer was added, the sample heated at 85°C to stop the reaction and the extent of substrate protein degradation was then assessed using SDS-PAGE (see section 2.4.1). An aliquot equivalent to 1.0 μg of the substrate protein was loaded per lane of the gel.

## 3 Results

### 3.1 Gelatinase assays

**Fig. 1:**
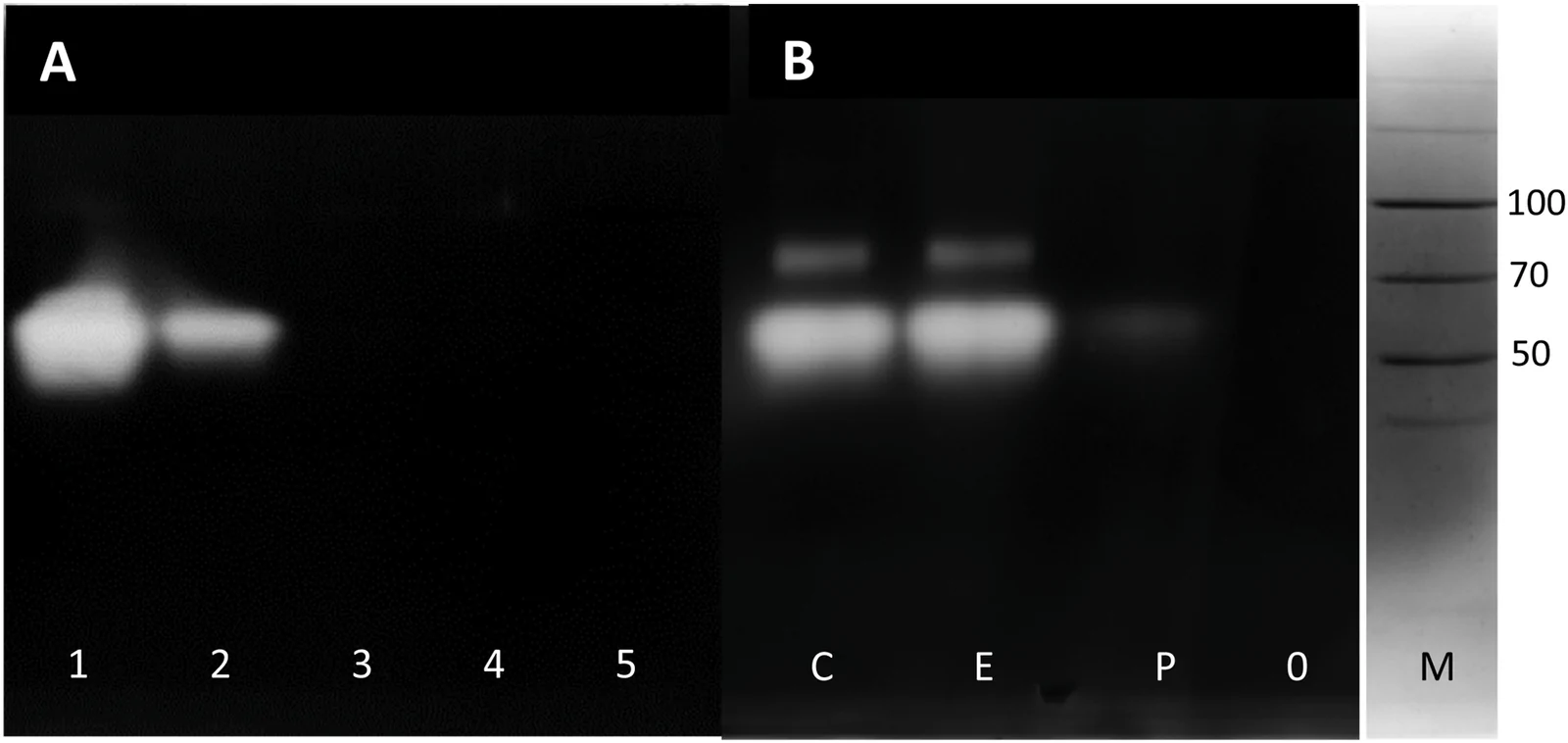
**Gelatinase activity (gelatin zymogram) of Nigerian and Tanzanian *B. arietans* venoms.** The zymograms were prepared with 0.2% (w/w) gelatin in a 10% acrylamide SDS-PAGE gel. The gels were run under non-reducing conditions and 2 μg of venom was loaded per lane. The zymograms were then incubated as in section 2.4.5.1 and then visualised by staining with Coomassie Blue R250. **A:** Zymogram of venoms: lanes 1, 2: NGA snakes 011 and 016; lanes 3, 4: TZA snakes 005 and 006; lane 5: control (no venom). **B:** Zymogram of NGA 011 venom treated with protease inhibitors. The venoms were pre-incubated with either EDTA or PMSF as described in the main text and then run as for zymogram A. Lanes C, controls (un-inhibited venoms); E, EDTA-treated venom; P, PMSF-treated venom; 0, no venom. Lane M contains molecular weight markers (Thermo Page Ruler, indicated in kDa) which were run under reducing conditions and visualised after fully destaining the gel.

Whilst studying the activities of the prominent PI SVMPs of puff adder venoms (Wilkinson et al., 2026), we observed a strong gelatinolytic activity in many venoms that was shown to be due to serine protease (SVSP) activity (Fig. 1A). The figure shows an example of two of each of venoms from the five Nigerian and four Tanzanian snakes kept at LSTM. The NGA venoms contained a strong gelatinase activity at around 55-60 kDa which was most active in NGA snake 011. No such activity was observed in any of our TZA venoms, not just the two shown here (see Fiig. S1A for further examples). The pronounced difference between the activity in the Nigerian and Tanzanian venoms seen in Fig. 1 is typical of the geographical variation observed using a wide range of puff adder venoms from stocks held at LSTM, which can be seen in Fig. S1. In these examples, venom from Eswatini and Kenyan snakes held at LSTM showed gelatinase activity as did four stock Nigerian venoms, and stock venoms from Ghana, Malawi, Zimbabwe and one South African venom. A second sample of South African venom and one from Saudi Arabia showed no gelatinase activity. Zymogram B shows the variation in gelatinase activity in venom from three different Kenyan puff adders held at LSTM: not only in the degree of activity, but differences in the levels of two different forms of the protein can also be seen.

The same assay format was used to measure the effects of protease inhibitors to determine the class of the protease responsible for the activity. EDTA or PMSF, inhibitors of SVMPs and SVSPs, respectively, were incubated with the most gelatinolytic NGA venom (Fig. 1B). The activity in this venom was inhibited by PMSF, but not EDTA, and therefore the 55-60 kDa gelatinases are of the serine protease class. Since gelatinase activity in vipers is more commonly found to be due to SVMPs, we set out to isolate and characterise the SVSP responsible for the gelatinase activity. The work was focussed on the Nigerian venoms because a full transcriptome was available for snakes from this region (Dawson et al., 2024).

### 3.2 Isolation and biochemical characterisation of the serine proteases (SVSPs)

**Fig. 2:**
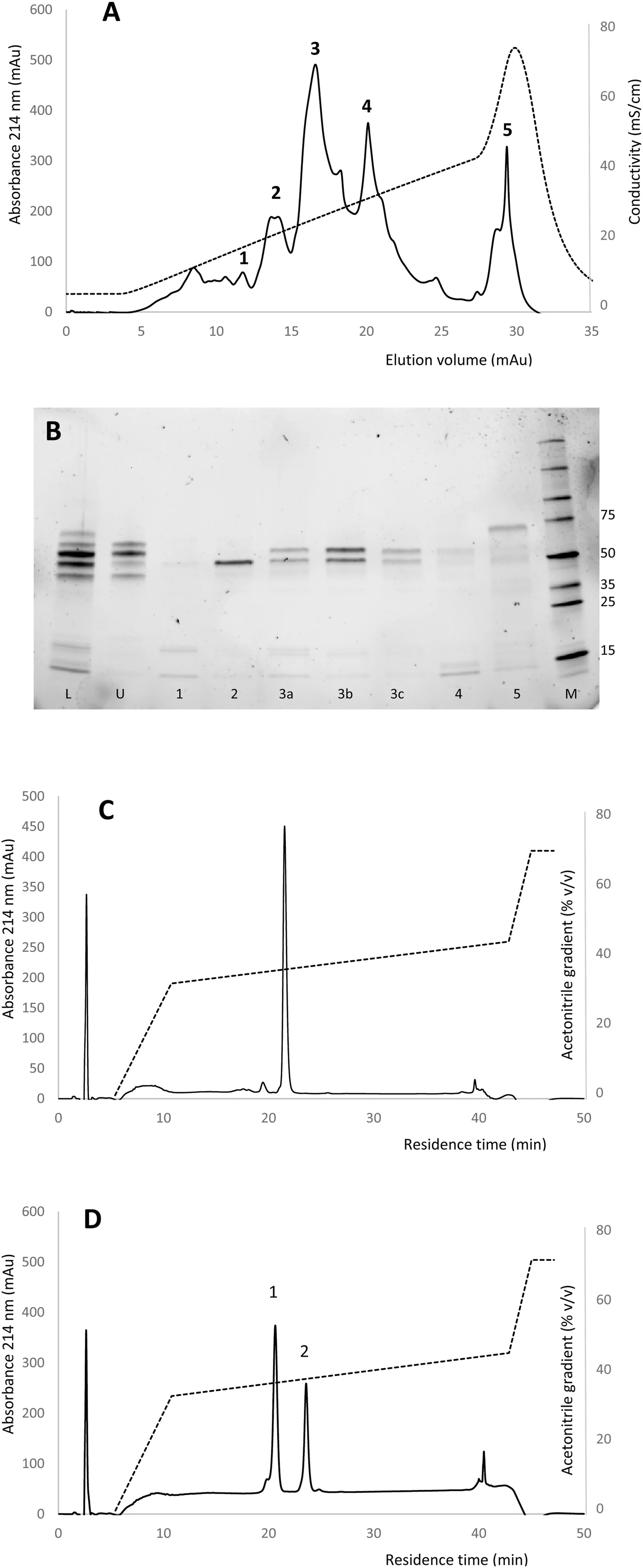
**Purification of the basic SVSPs from NGA SEC peak 2. A:** The proteins in SEC peak 2 (Fig. S2) were applied to a 1 mL HiRes Capto S column equilibrated in 50 mM sodium phosphate pH 5.2. Elution was carried out using a gradient of 0 - 0.7 M NaCl and monitored for absorbance at 214 nm (solid line) and conductivity (dashed line). The proteins were loaded onto the column independently of the program used to perform the chromatography, so the peak of unbound protein used for the subsequent anion exchange chromatography (Fig. 3) is not visible here**. B:** SDS-PAGE of the main fractions from the cation exchange run. L, load; U, unbound proteins; 1-5 fractions from the respective chromatography peaks (peak 3 was run as three fractions a-c collected from across the peak); M, markers (Promega Broad Range) with molecular weights of key markers indicated in kDa. The gel was 4-20% acrylamide, stain-free (BioRad). **C** and **D:** C4 RP-HPLC (see Sec. 2.3) of the main proteins from cation exchange chromatography, C, peak 2; D, peak 3b. A gradient of acetonitrile was used for elution (dashed line) and absorbance was monitored at 214 nm (solid line).

Following SEC of NGA venoms, the second elution peak (Fig. S2 peak 2) contained multiple proteins in the 50-65 kDa size range and was rich in protease activity. Neither EDTA or PMSF could fully inhibit this activity, indicating that there are both SVSPs and SVMPs in this peak. The SVSP gelatinase activity observed in the whole NGA venom (Fig. 1A) was also found here, so this venom was used to develop isolation methods for the individual proteases in the 50-65 kDa size range. The material from this peak was applied to a cation exchange chromatography column (see Sec. 2.3). This resulted in a large amount of material passing through unbound as well as material which bound and then eluted in the NaCl gradient in multiple peaks (Fig. 2A). Peak 2 contained a single 52 kDa protein and peak 3 contained proteins at 52 and 56 kDa (Fig. 2B). RP-HPLC was used to separate the individual SVSPs and, following removal of the solvent and resuspension in PBS, their protease activity was found to have been retained. This RP-HPLC step enabled a full separation of the two proteins in cation exchange chromatography peak 3 (Fig. 2D, peak 1 is 56 kDa; peak 2 is 52 kDa). These two proteins and that from cation exchange peak 2 will be referred to henceforth as basic SVSPs.

**Fig. 3:**
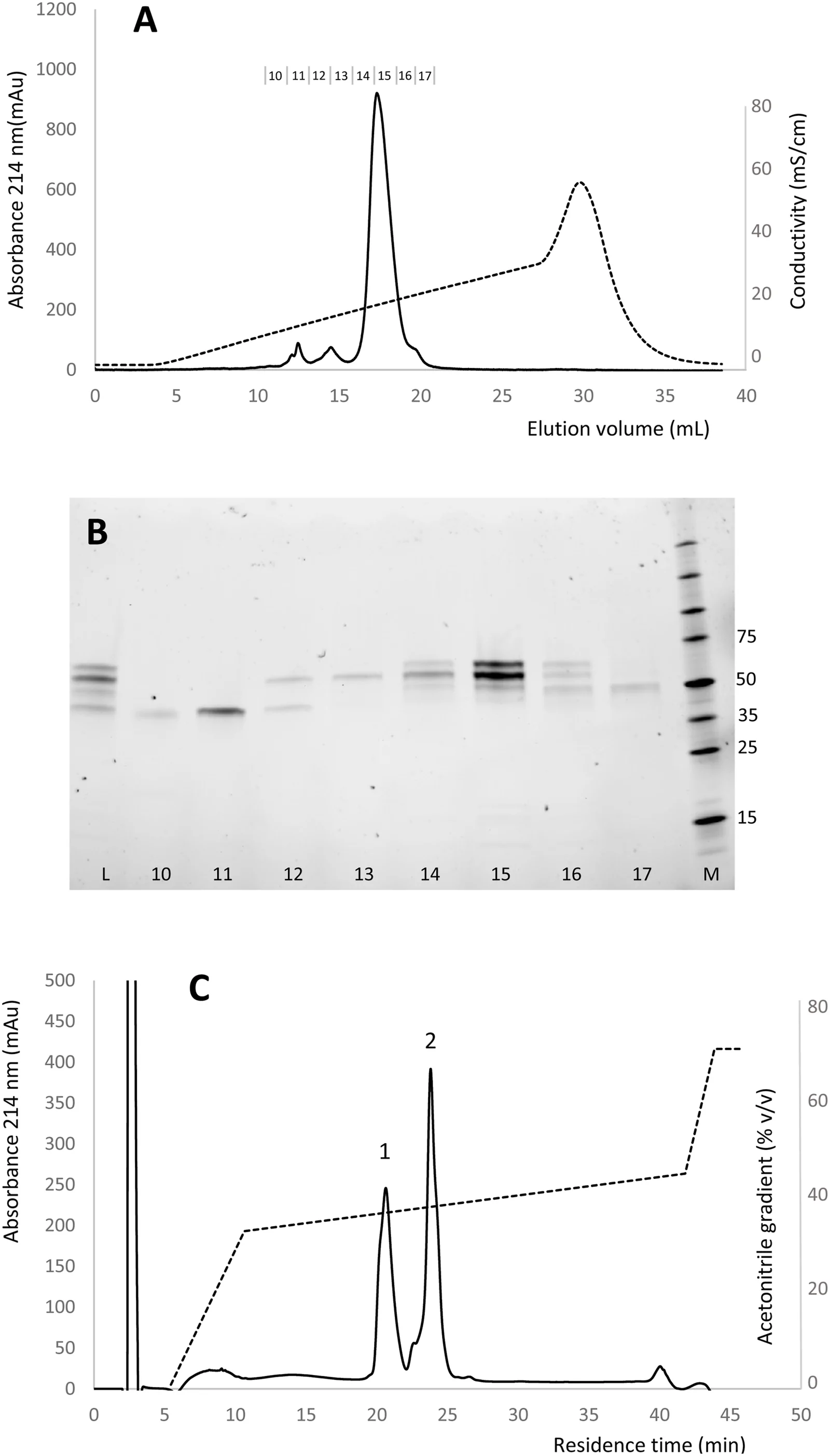
**Purification of the acidic SVSPs from NGA SEC peak 2. A**: The unbound material from the cation exchange step (Fig. 2) was dialysed against 50 mM Tris-Cl, pH 8.5 and loaded onto a 1 mL Mono Q column equilibrated in the same buffer. The acidic SVSPs were eluted from the column using a gradient of 0-0.4 M NaCl and elution was monitored for absorbance at 214 nm (solid line) and conductivity (dashed line). **B**: SDS-PAGE of the main fractions (numbers indicated in the inset of panel 3A) from the cation exchange run. L, load; 10-17, fractions from around the main chromatography peaks; M, markers (Promega Broad Range), molecular weights of key markers are indicated in kDa. The gel was 4-20% acrylamide, stain-free (BioRad). **C:** C4 RP-HPLC (see Sec. 2.3) of anion exchange chromatography peak 3. A gradient of acetonitrile was used for elution (dashed line) and absorbance was monitored at 214 nm (solid line).

The proteins that did not bind to the cation exchange column (see SDS-PAGE lane U, Fig. 2B) also possessed SVSP activity. These were dialysed against 50 mM Tris-Cl, pH 8.5 and subjected to anion exchange chromatography. The bulk of the bound protein eluted in one main peak (peak 3, Fig. 3) which contained prominent bands at 58 and 62 kDa. An earlier-eluting peak (1) contained a band at 43 kDa. The proteins in anion exchange chromatography peak 3 were subject to RP-HPLC (Fig. 3C) which resulted in a good separation of the two main proteins observed on SDS-PAGE (Fig. 3C, peak 1 is 60 kDa; peak 2 is 56 kDa). As in the case of the basic SVSPs, both retained their protease activity following this final isolation step.

**Fig. 4:**
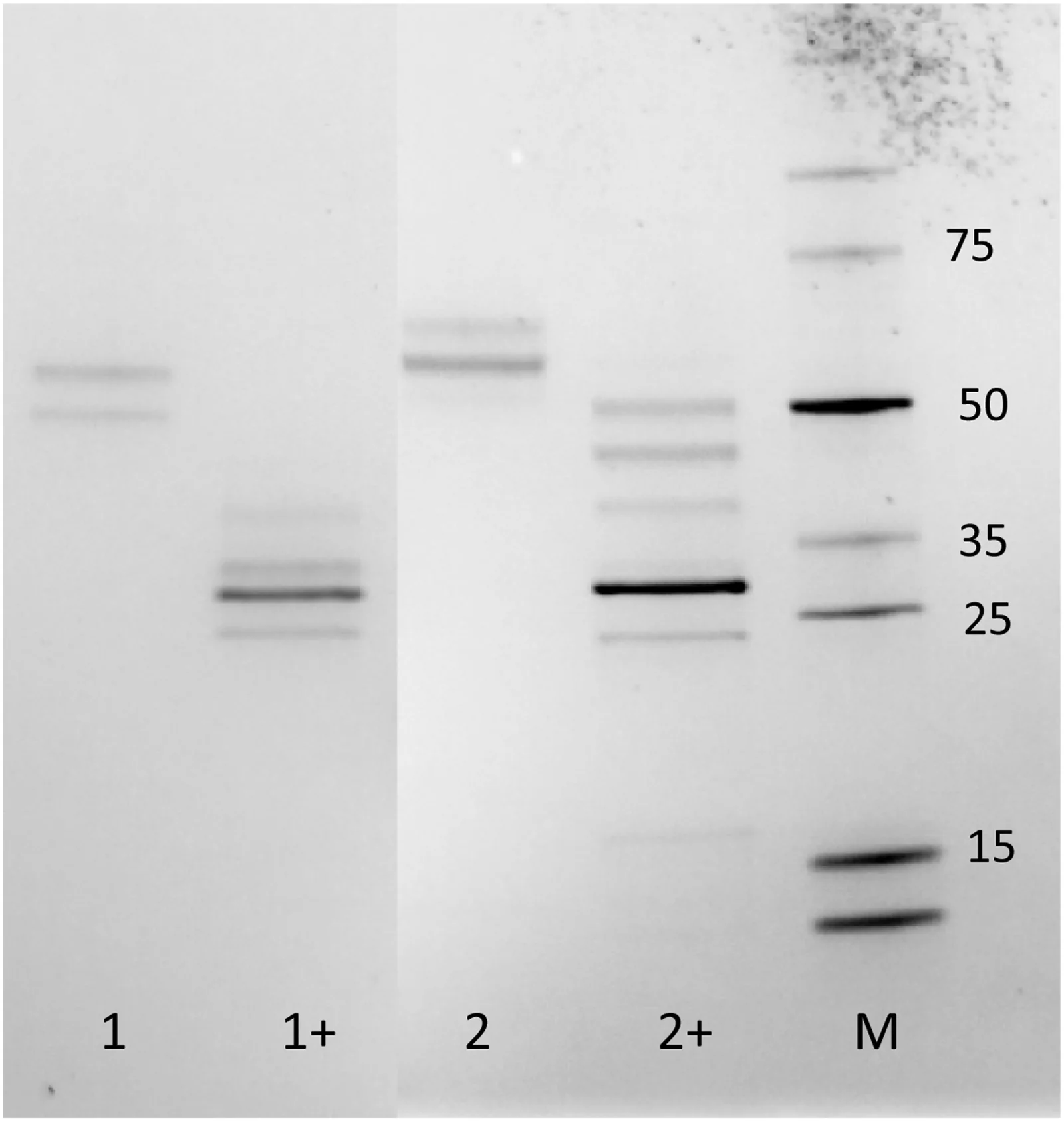
**De-glycosylation of the pure SVSPs**. Samples were denatured then treated with PNGase F for 3 h at 42°C. Lanes 1, 1+, basic SVSPs; lanes 2, 2+, acidic SVSPs. Lanes indicated + are the PNGase F-treated proteins. The prominent band at around 30 kDa in these lanes is PNGase F. The molecular weights of the markers (Promega Broad Range) are indicated in kDa on the right.

PNGase F treatment was used to determine the number of N-glycans on the basic and acidic SVSPs (Fig. 4). The purified proteins in the main ion exchange peaks were used for this analysis. In both cases, glycan cleavage was incomplete, but this allowed visualisation of intermediates with the full range of N-glycans. For both proteins, the lowest molecular weight band was around 24 kDa, a size that would be predicted from the sequence. From there upwards (increasing molecular weight) and compiling the results from all 4 lanes on the gel, bands can be seen in 2-3 kDa steps which would account for the protein chain plus 1 (just above the PNGase F band at ∼30 kDa), 2, 3, 4 (alongside the 50 kDa marker), 5 and 6 N-glycans. This would suggest that the two bands in the basic SVSP peak have 4 (the 52 kDa form) and 5 (the 56 kDa form) N-glycans, and that of acidic SVSPs, 5 (the 56 kDa form) and 6 (the 60 kDa form) N-glycans.

**Fig. 5:**
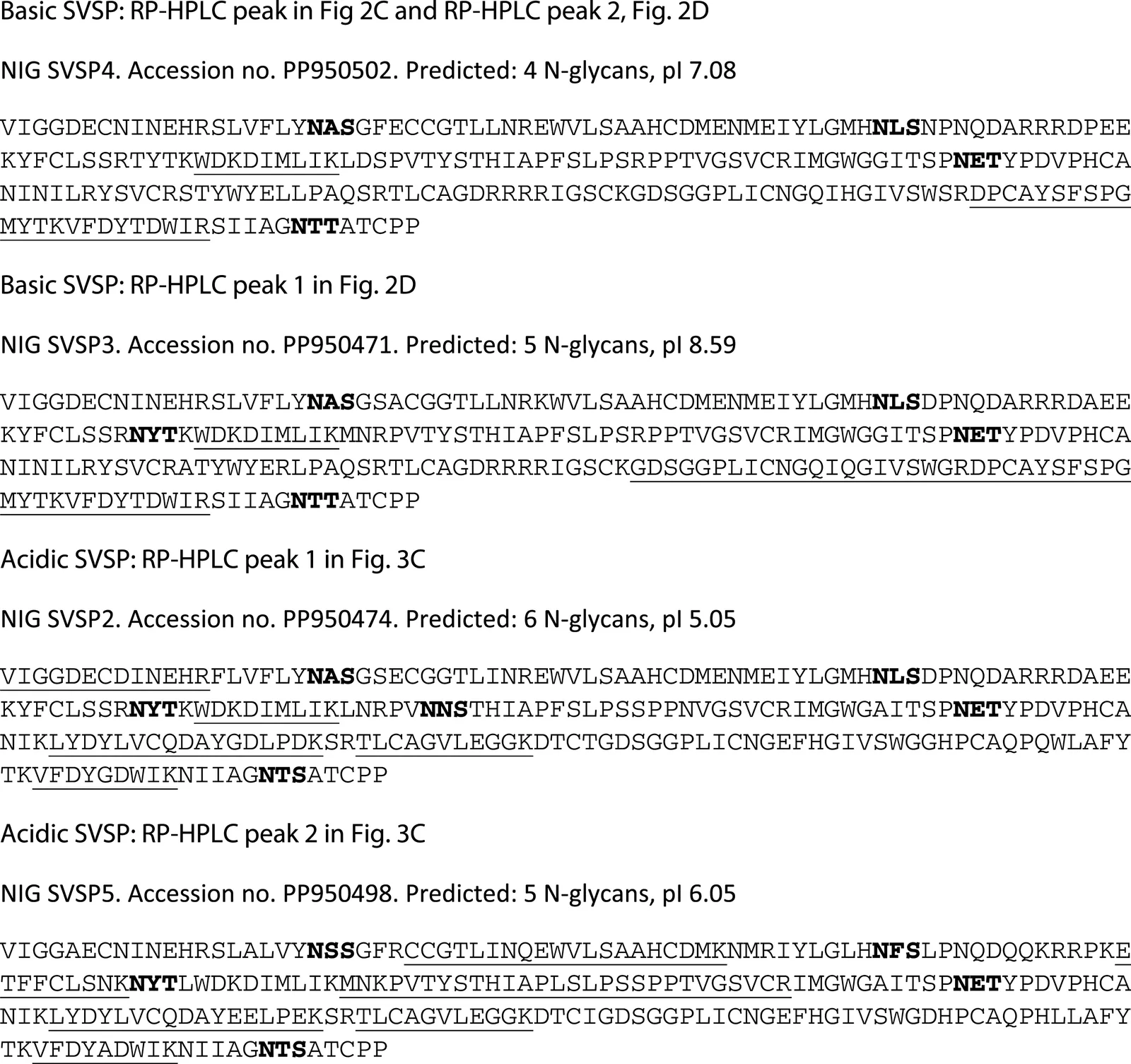
**Amino acid sequences of the transcripts with best matches to the SVSPs isolated from NGA venoms.** Ten μg of the pure proteins was digested with trypsin and the resulting peptide mix was analysed by RP-HPLC-MS/MS (Sec. 2.4.3). Fragmentation spectra were searched against an in-house *B. arietans* (NGA) venom gland derived protein sequence database using Mascot. Identified peptides are underlined (full MSMS data can be found in the supplementary material). Predicted N-glycan sites in the sequence are indicated in bold font.

The RP-HPLC purified SVSPs were digested with trypsin and subjected to MS/MS. The resulting data was searched against a list of 24 SVSPs sequences identified in the transcriptome of an NGA snake (Dawson et al., 2024). The best matches are shown in Fig. 5 (MS/MS data can be found in the supplementary material). The predicted pI and number of N-glycans matches well, respectively, with the chromatographic behaviour and the number of N-glycans determined experimentally. The N-glycan number has been used help to assign basic SVSP4 sequence to the proteins indicated, since the tryptic peptides found also match to SVSP3. Although there is much homology between the basic and acidic forms, there are critical differences in the substrate binding sites (see Discussion).

### 3.3 Confirmation of protease class and determination of peptide bond specificity of the isolated SVSPs

**Fig. 6:**
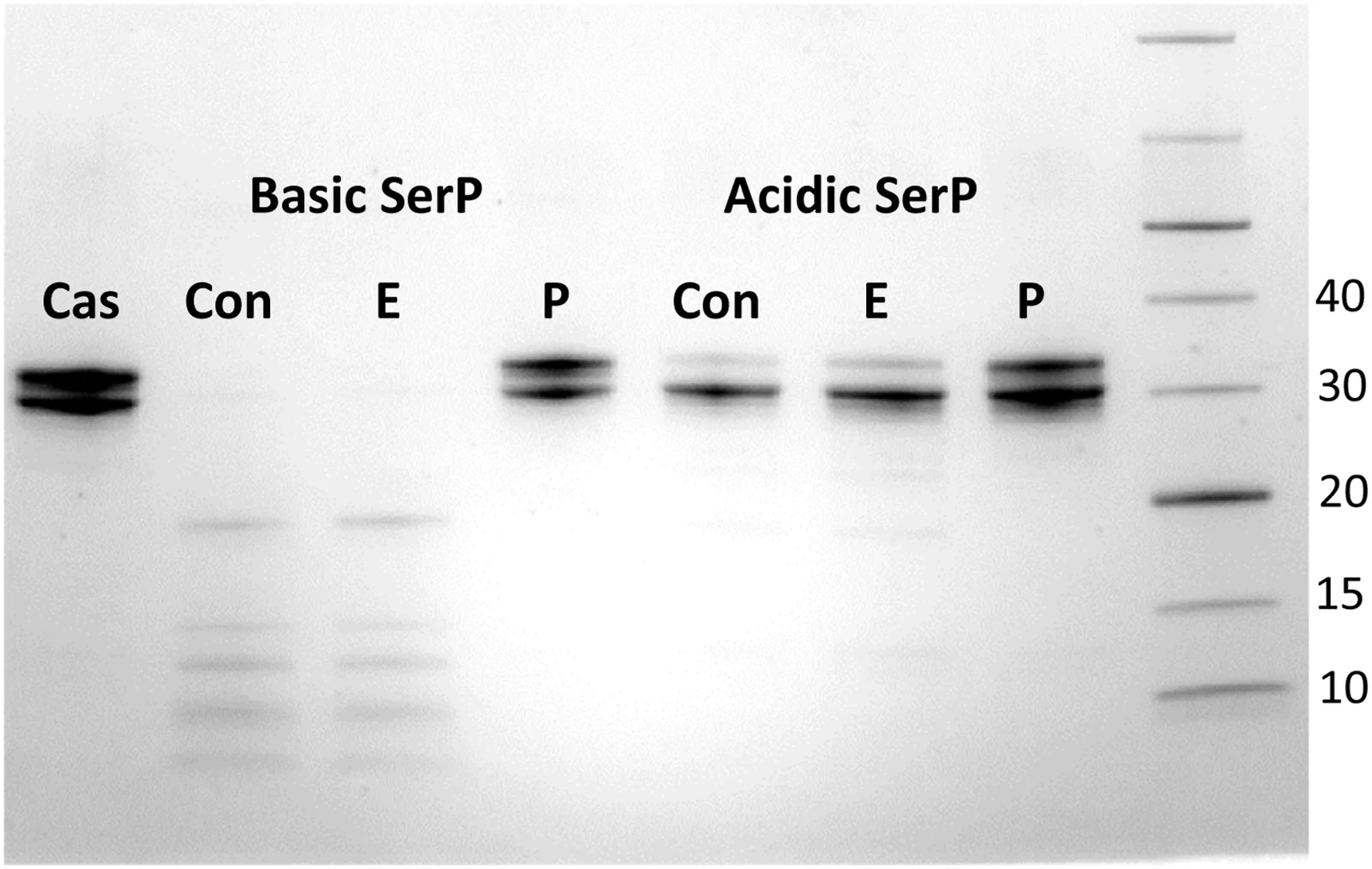
**Casein degradation assay: Purified serine proteases from NGA puff adder.** Basic or acidic SVSPs from the ion exchange chromatography steps were incubated with bovine casein, either alone or with 5 mM EDTA or 2 mM PMSF, for 90 min at 37°C. An aliquot of the reaction mix equivalent to 2 μg of casein was run on SDS-PAGE. The gel used was 4-20% acrylamide (BioRad) and was stained with Coomassie Blue R250. Cas, casein control; Con, SVSP with casein; E, SVSP + casein + 5 mM EDTA; P, SVSP + casein + 2 mM PMSF. Molecular weight markers (Thermo Page Ruler) are indicated in kDa on the right. (For interpretation of the references to colour in this figure legend, the reader is referred to the Web version of this article.)

**Fig. 7:**
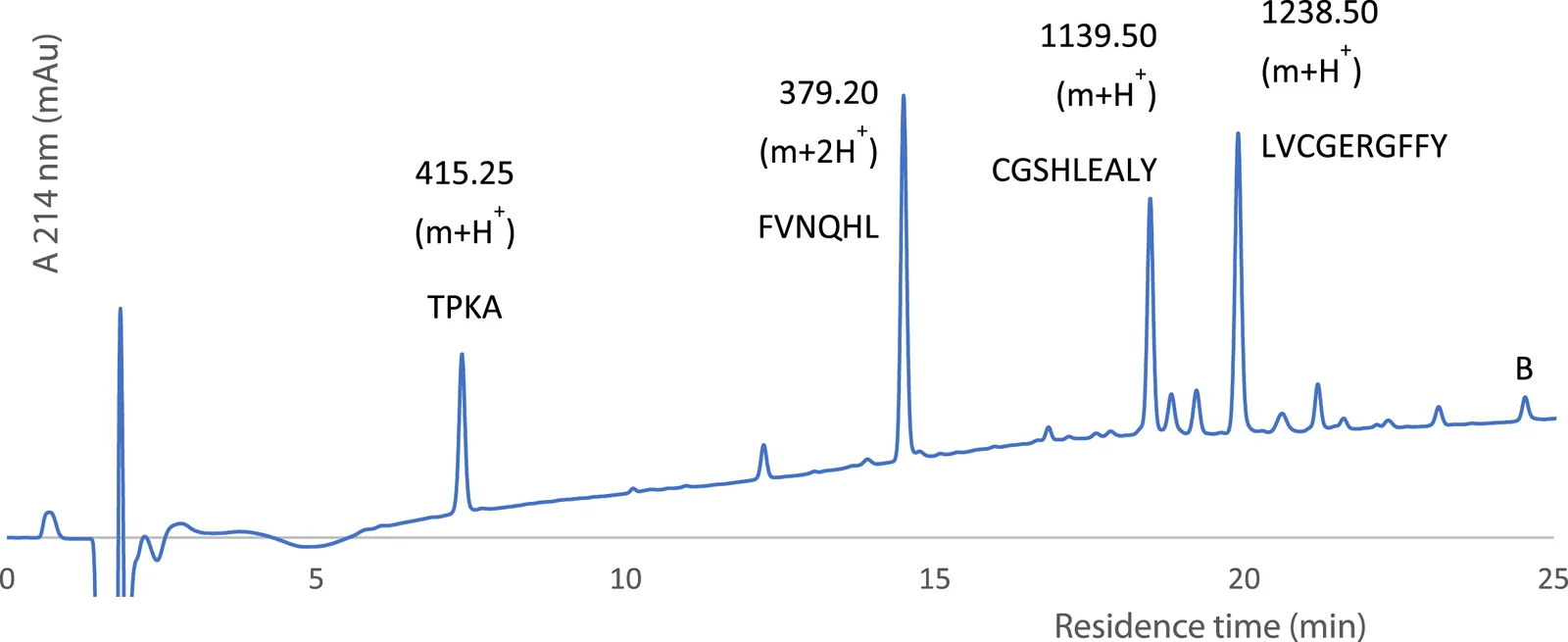
**Insulin B degradation assay: Purified basic SVSP.** The main basic (RP-HPLC peak 1 in Fig. 2D) SVSP from the ion exchange chromatography step was incubated at 37°C for 120 min with insulin B chain at a ratio of 20:1 (w/w) insulin B: SVSP. An aliquot containing the equivalent of 1 μg insulin B was analysed by RP-HPLC with monitoring at 214 nm (top figure). The insets show the mass values obtained using LC-MS (see also Table S3). B, remaining intact insulin B chain. Below the chromatography trace the products from insulin B digestion are shown relative to the intact peptide.

Using a casein assay with degradation measured using SDS-PAGE, the basic and acidic SVSPs were shown to be inhibited by PMSF but not EDTA (Fig. 6). The acidic SVSPs were not as active towards casein as were the basic SVSPs. Using insulin B chain as protease substrate with RP-HPLC-UV analysis also revealed a distinct difference between the activity of the two SVSP forms. The acidic SVSPs were unable to cleave any of the peptide bonds of insulin B whereas four main degradation products were seen with the basic SVSP (Fig. 7), which were not greatly altered when the protease was incubated with EDTA, but were absent when PMSF was used for pretreatment (results not shown).

**Fig. 8:**
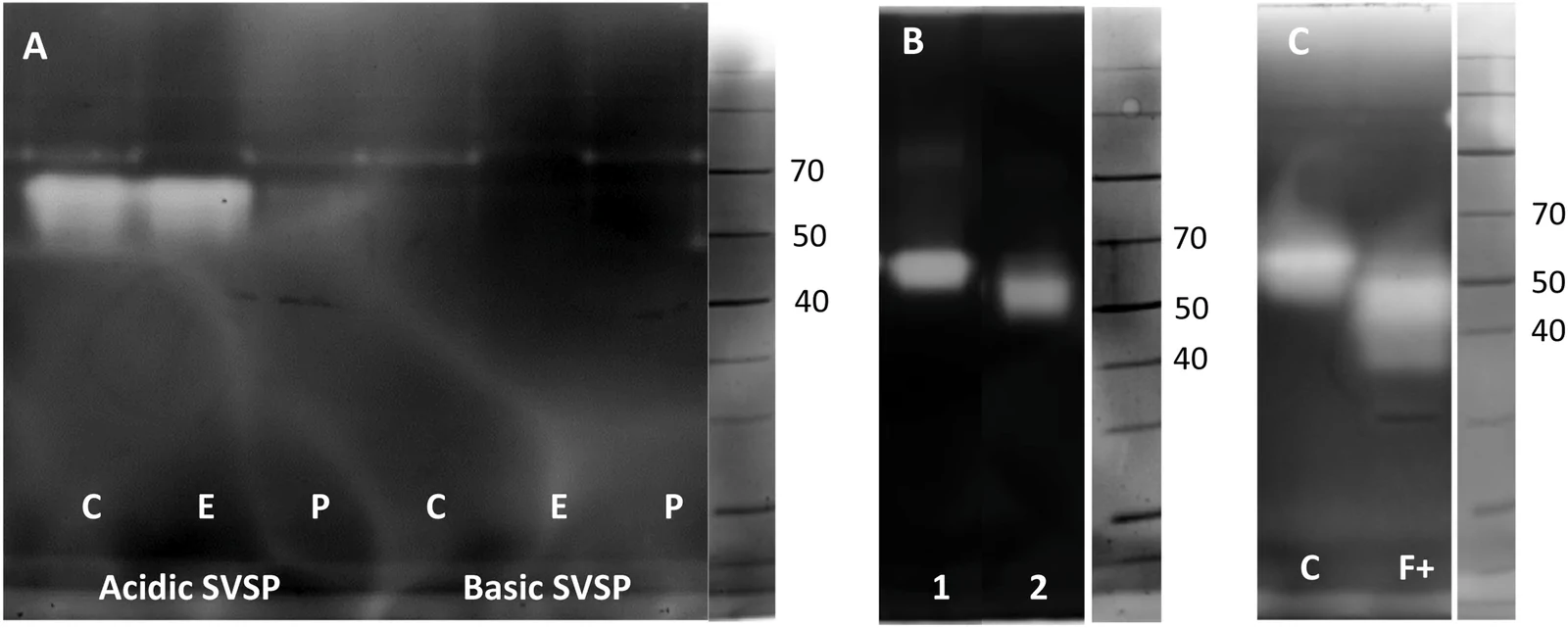
**Gelatinase activity (gelatin zymograms) of the SVSPs from NGA *B. arietans* venoms.** The zymograms were prepared with 0.2% (w/w) gelatin in a 10% acrylamide SDS-PAGE gel, which was run under non-reducing conditions. The zymograms were then incubated as in section 2.4.4.1 and then visualised by staining with Coomassie Blue R250. **A**: purified acidic and basic proteases (0.1 μg) with inhibitors. C, control (no inhibitor), E, 10 mM EDTA; P, 2 mM PMSF. **B**: Proteins (0.5 μg) from peak 1 (lane 1) and 2 (lane 2) of RP-HPLC of the acidic SVSPs (see Fig. 3C). **C.** Effect of deglycosylation on the gelatinase activity of the acidic SVSP (0.2 μg of peak 1 from RP-HPLC, Fig. 3C). Lane C, control, no PNGase F; lane F+, PNGase F treated. On the right of each zymogram is the lane containing Thermo Page Ruler molecular weight markers (kDa) run on the same gel and visualised after extensive destaining.

The products of the reaction of the basic SVSP (RP-HPLC peak 1 in Fig. 2D) with insulin B chain were analysed by RP-HPLC-MS and (see insets Fig. 7 and full details in Table S3). The major products have arisen from digestion at the L-C, Y-L and Y-T peptide bonds in insulin B. The other main form of basic SVSP (RP-HPLC peak in Fig. 2C and RP-HPLC peak 2, Fig. 2D) cleaved at essentially the same sites, but was less able to fully cleave the Y-T peptide bond.

A set of chromogenic esterase substrates were also used to determine the substrate site (P_I_) specificities of the purified serine proteases (Table 1). The acidic SVSPs showed activity against just the arginine-containing BAEE substrate, confirming them to be trypsin-like SVSPs. The basic SVSPs had no activity against BAEE, nor towards the tyrosine-containing BTEE substrate traditionally used to determine chymotrypsin-like SVSP activity. Using a set of nitroanilide esters with Val, Leu, Ala and Phe in the key P_I_ site, the basic SVSPs showed a small but definite activity towards that containing phenylalanine (N-succinyl-A-A-P-F-pNA). The acidic SVSPs (and trypsin) were active against both of the Chromogenix substrates commonly used to identify general trypsin-like (S-2288) and kallikrein-like (S-2302) serine protease activities.

**Table 1:** Activity of NGA SVSPs towards standard serine protease substrates.

*Enzyme*	Specific activity (μmol product formed/min/mg protease)							
	Ethyl esters		Nitroanilide (p-NA) esters					
	*BAEE*	*BTEE*	*Ala*	*Leu*	*Val*	*Phe*	*S-2288*	*S-2302*
Trypsin	36.5	nd	nd	nd	nd	0	10.8	2.12
Chymotrypsin	nd	27.3	0	1.85	0	12.2	nd	nd
Elastase	nd	nd	2.50	8.56	1.83	0	nd	nd
Acidic SVSP	7.13	0	0	0	0	0	0.901	0.896
Basic SVSP	0	0	0	0	0	0.031	0	0

Each result is the average of three determinations. The basic SVSP used was a pooled fraction of the 52 and 56 kDa RP-HPLC peaks (see Fig. 2D). The acidic SVSP used was from anion exchange peak 3b (see Fig. 3). nd = not determined. The chromogenic substrates S-2288 and S-2302 both contain Arg esterified to p-NA.

### 3.4 Activity of the isolated proteases in the functional assays

#### 3.4.1 Gelatinase activity

The studies using whole venom in gelatin zymogram assays showed that the gelatinase activity in the NGA venoms was due to the action of serine proteases (see Fig. 1). Using the purified NGA SVSPs in the same assay it became clear that this activity resides solely with the acidic SVSPs (Fig. 8A). The activity of the latter was not inhibited by EDTA but was so by PMSF. The basic SVSPs demonstrated no gelatinase activity. Both RP-HPLC-purified 56 and 60 kDa acidic SVSPs were active (Fig. 8B), with gelatin-degrading activity observed at their respective molecular weights. An attempt was made to determine the importance of the N-glycans to the gelatinase activity (Fig. 8C). Deglycosylation was carried out under native conditions, but this resulted in an incomplete removal of the N-glycans; SDS-PAGE analysis appeared to show the loss of just 2 of the N-glycans (result not shown). The partially deglycosylated material appears to have lost no gelatinase activity, however (Fig. 8C, lane F+).

#### 3.4.2 Fibrinogen, prothrombin and laminin degradation

**Fig. 9:**
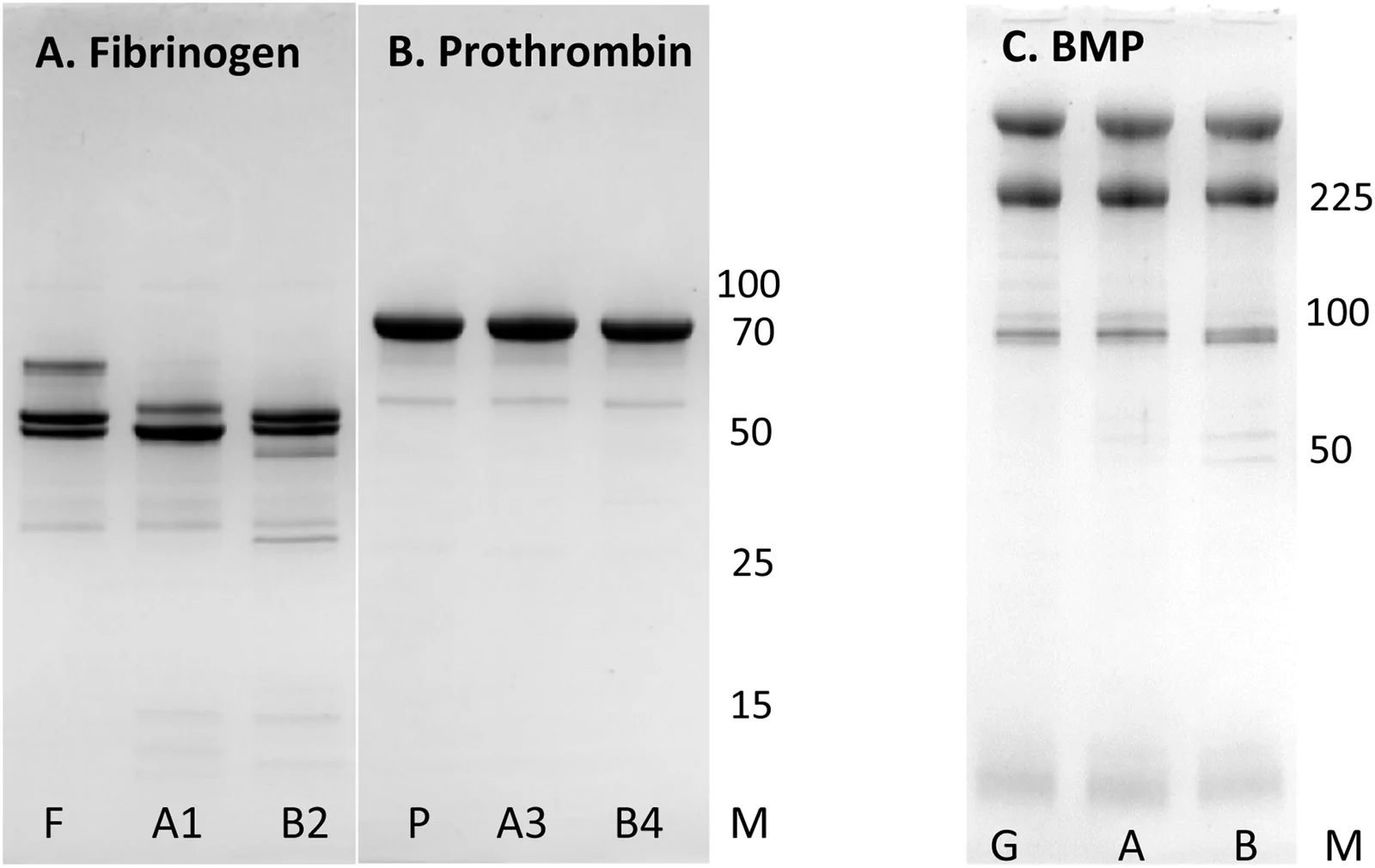
**SDS-PAGE analysis of the degradation of fibrinogen, prothrombin and laminins (basement membrane proteins) by purified *B. arietans* SVSPs.** The gels were run under reducing conditions using 4-20% acrylamide (BioRad) and were stained using Coomassie Blue R250. The molecular weights of key markers (lane M, Promega Broad Range) are indicated in kDa. **A** and **B: Fibrinogen** (gel A) or **prothrombin** (gel B) were incubated at 37°C for 120 min with the proteases at a 30:1 (w/w) ratio of substrate:SVSP. The equivalent of 1.0 μg of substrate protein fibrinogen (F) or prothrombin (P) was loaded per lane. Lane F is fibrinogen control and lane P is prothrombin control; lane A1, F + acidic SVSP; lane B2, F + basic SVSP, lane A3, P + acidic SVSP; lane B4, P + basic SVSP. **C: Laminins**. A basement membrane protein (BMP) extract (Geltrex) was incubated at 37°C for 30 min with the purified SVSPs at a 30:1 (w/w) ratio of BMP:SVSP. The equivalent of 8 μg of BMP was loaded per lane. Lane G, BMP control, the two prominent bands >225 kDa are alpha and beta laminin; lane A, NGA acidic SVSP + BMP; lane B, NGA basic SVSP + BMP.

The purified SVSPs were tested for proteolytic activity against the key plasma proteins commonly targeted by haemotoxic venom proteases: fibrinogen and prothrombin, and also against the laminins in basement membrane protein extract (Fig. 9). The NGA basic and acidic SVSPs used for this analysis were the RP-HPLC purified proteins from the main ion exchange peaks (Fig. 2A and 3A). Both acidic and basic SVSPs were able to digest fibrinogen to some degree but the degradation pattern for the acidic SVSP (Fig, 9A, lane A1) differs from that of the basic (lane B2). The acidic SVSP showed limited cleavage of both the alpha band, to a position just above the beta band in the control lane, and the beta band to the same position as the gamma band. The basic SVSP appears to possess alpha-fibrinogenase activity, with no apparent degradation of the beta and gamma bands. Despite using the same experimental conditions that are routinely used in our laboratory to successfully demonstrate activation of prothrombin or degradation of laminins by purified viper proteases, neither form of SVSP showed any proteolytic activity towards either of these substrates (Fig. 9B and C respectively).

#### 3.4.3 Cleavage of angiotensin-related peptides

**Fig. 10:**
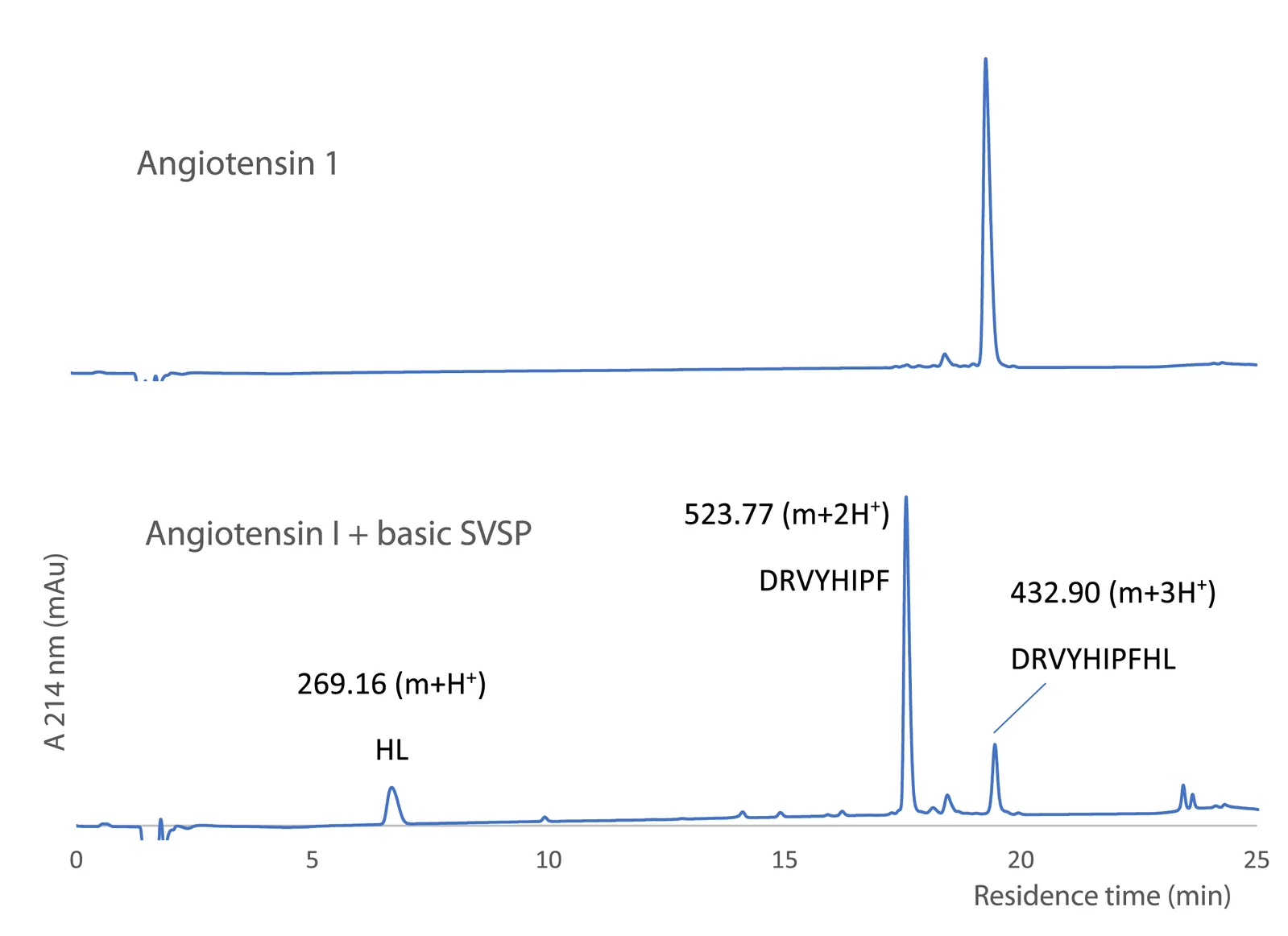
**Action of the basic SVSP on angiotensin I (1-10).** The basic SVSPs from the cation exchange chromatography step was incubated at 37°C for 60 min with angiotensin I at a ratio of 30:1 (w/w) angiotensin I:SVSP. An aliquot containing the equivalent of 1 μg angiotensin I was analysed by RP-HPLC with monitoring at 214 nm. The results of RP-HPLC-MS analysis can be seen in Table S4.

A previous study of *B. arietans* (Paixão-Cavalcante et al., 2015) had shown that whole venom could convert angiotensin I (1-10, DRVYIHPFHL) to the vasodilator angiotensin 1-7 (DRVYIHP) and this was recently shown to be due to a PI SVMP found in Tanzanian and Nigerian puff adder venoms (Wilkinson et al., 2026). Because of the pronounced differences in the levels of SVSP in these two regional venoms, the action of the basic and acidic SVSPs on angiotensin I was determined. The acidic SVSPs did not cleave angiotensin 1, but the basic form cleaved this decapeptide into two main peptide products detected by RP-HPLC-UV analysis (Fig. 10). RP-HPLC-MS analysis showed that the acidic SVSP had specifically cleaved the F-H bond of angiotensin I, generating the vasoconstrictive peptide angiotensin II (ang 1-8, DRVYIHPF) plus the dipeptide HL (Table S4). The basic SVSP also acted on a peptide homologous to residues 1-14 of porcine angiotensinogen (DRVYIHPFHLLVYS), a precursor to angiotensin I. It cleaved at three consecutive sites in the latter (F-H-L-L) generating angiotensin I (1-7), angiotensin 1-8 (DRVYIHPF) and angiotensin 1-9 (DRVYIHPFH, a vasodilator) and the C-terminal peptide HLLVYS (see Table S4). Since the acidic SVSP and the PI SVMP are both present in some puff adder venoms and may compete in their action on angiotensin I, the action on the latter by whole venoms was determined. The Tanzanian venom, which contains the PI SVMP but little SVSP, generated angiotensin 1-8 only, whereas the Nigerian venom used here, which has both the PI SVMP and the basic SVSP, generated a mixture of the products of the action of both proteases: angiotensins 1-8 and 1-7 respectively (Table S4).

## 4 Discussion

A complete set of SVSPs was isolated from Nigerian puff adder venoms which had previously been shown to be rich in this class of protease. During this procedure, these SVSPs separated into two groups: basic and acidic forms. Despite their similarity in molecular weights and in their degree of N-glycosylation, each group possessed distinct enzymatic activities towards a variety of general protease substrates. Differences were observed between the ability of the two forms to degrade casein, with the basic form apparently acting with a greater potency. This difference in activity was most pronounced towards small peptide substrates, insulin B chain and angiotensin: basic SVSPs cleaved both of these at multiple sites, but the acidic forms were unable to cleave any of the bonds these peptides. Using small chromogenic substrates, the acidic SVSPs were shown to be trypsin like, but the basic SVSPs were clearly non-trypsin-like and were active only against a phenylalanine-containing substrate. Mass spectrometric analysis of the products of insulin B chain and angiotensin digestion by the basic SVSPs also supported the findings from the chromogenic substrates, cleaving peptide bonds C-terminal to tyrosine, leucine and histidine as well as phenylalanine. A bioinformatic analysis of SVSP sequences in a Nigerian puff adder transcriptome supported these findings and this will be expanded upon in this section.

Differences in the activity of the basic and acidic SVSPs towards proteins of potential functional and pathological significance was observed, the most interesting of which was in their ability to degrade gelatin. The acidic SVSPs were found to be responsible for the strong gelatinase activity observed in NGA venoms, whereas the basic SVSPs possessed no such activity. Gelatinase activity in venoms is usually associated with the SVMPs and such activity has not been previously determined in an isolated SVSP, which up to now have been shown to act only on plasma proteins (see Serrano, 2013). There is evidence in the literature for such activity, however: a PMSF-inhibited 36 kDa gelatinase was observed in zymograms of *B*. *nasicornis* whole venom (Paixão-Cavalcante et al., 2015). Such activity, at around 50-60 kDa, was also observed in 11 of 12 NGA B*. arietans* tested venoms in an earlier study (Currier et al., 2010), but was reasonably assumed to be due to the action of an SVMP, since such activity would be unexpected for a SVSP based on the knowledge at the time. SVSP gelatinase activities may be peculiar to the large *Bitis* spp.

**Fig. 11:**
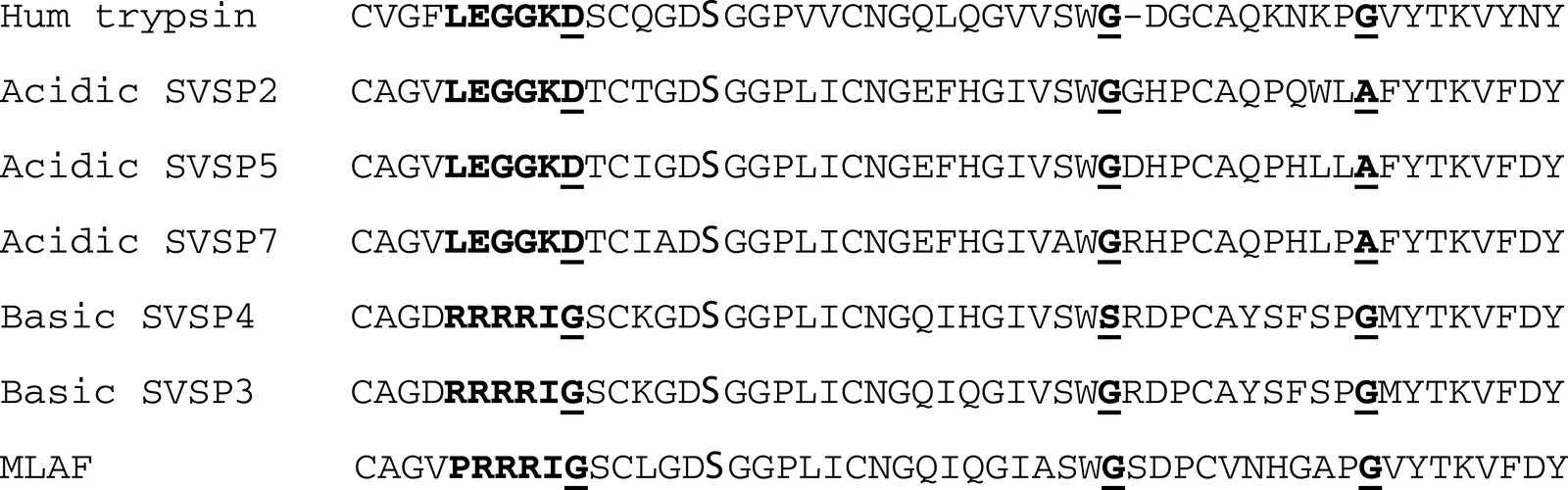
**Sequence alignment of the substrate-binding region of the most abundant acidic and basic SVSP transcripts from NGA puff adder**. The four NGA SVSPs sequences (acidic SVSP 2 and 5, basic SVSP 3 and 4, see Fig. 5) were subject to a CLUSTAL multiple sequence alignment by MUSCLE 3.8 (Larkin et al., 2007). Also included for comparison are sequences from human trypsin-1 (P07477), *M. lebetina* alpha-fibrinogenase MLAF (Q8JH85 VSPA_MACLB) and acidic SVSP7 which has tryptic peptide matches to the 33 kDa kinin-releasing SVSP Kn-Ba isolated by Megale et al. (2018). In bold is the S_1_ residue (D189 or G189) plus the 5 residues immediately N-terminal to this. The active site S195 is shown in bold/italics. Shown in bold/underlined are the three key substrate pocket residues 189, 216 and 226 (DGG in trypsin; DGA in the acidic SVSPs; GGG or GSG in basic SVSPs and GGG in MLAF). All residues are numbered using the chymotrypsin system.

The availability of a full set of transcriptome data for Nigerian and Tanzanian snakes allowed us to match the biochemical characteristics of the purified SVSPs to predicted primary structures. An analysis of the NGA puff adder transcriptome found it to contain 24 different SVSP gene sequences, but only 6 were found in that of TZA snakes, and all with a very low transcripts-per-million score, mirroring the quantitative results of Dawson et al. (2024). Within the Nigerian set there is a broad range of predicted isoelectric points, ranging from 5.1 to 8.7 (not including a possible contribution from sialic acids of the N-glycans) but a full analysis of the sequences indicated the presence of two distinct groups of acidic and basic proteases, mirroring their chromatographic performance. The numbers of predicted N-glycans also matched the experimentally determined values: all the basic forms are predicted to have 4 or 5 N-glycans and the acidic forms 5 or 6 N-glycans, except for one acidic form with just 3 predicted N-glycan sites. More significant than these two features, however, is the basis for the differences in bioactivity which can be seen on analysis of their respective substrate-binding sites. Fig. 11 shows the relevant sections of the four NGA *B. arietans* SVSP transcripts which matched to the SVSPs isolated here.

In the case of the acidic SVSPs, the transcriptome data supports the experimental findings (Table 1) in that they are trypsin-like serine proteases, having an aspartate (**D** in Fig. 11) at the bottom of the substrate pocket (S_1_ binding site, residue 189 using chymotrypsin numbering). They also have sequences immediately N-terminal to the substrate-binding site (VLEGGKDT) similar to those of other well-studied SVSPs, such as batroxobin (Itoh et al., 1987). Thus, in most respects, these *B. arietans* acidic SVSPs are typical snake venom serine proteases (Serrano, 2013), though no others have so far been shown to have the gelatinase activity of the acidic SVSPs isolated here. At the two other positions that form the substrate pocket, 216 and 226, the acidic SVSPs have glycine (G) and alanine (A) residues respectively. The alanine at position 226 in lieu of the smaller glycine residue which is found here in trypsin and also in thrombin might restrict access of large residues in the P_I_ position of the substrate. Despite this, however, it appears to act on both alpha and beta fibrinogen in a similar manner to thrombin (Fig. 8A), which is quite rare for an SVSP (Kini, 2005).

It is difficult to explain the inability of the trypsin-like acidic SVSP to cleave at the lysine (K) and arginine (R) residues in insulin B and angiotensin I. Despite their being different classes of protease, it is interesting to note that both the puff adder SVMP PIII-c gelatinase isolated previously (Wilkinson et al., 2026) and the acidic SVSP gelatinase described here are unable to degrade insulin B, which is routinely used as a venom protease substrate because of its wide variety of peptide bonds. This all suggests that gelatinase activity in venom proteases is quite specific, perhaps with a primary specificity to amino acids at both P_1_ and P′_1_. This may point to a genuine functional role and it not being simply a case of a protease acting against a general protein substrate, such as is the case for other commonly used protease substrates, e.g. casein. Because of the possible functional and clinical relevance of the ability of a venom protease to degrade gelatin (predominantly denatured collagen I, which is rich in K and R residues) these SVSP and SVMP gelatinases warrant further studies beyond that of a simple biochemical interest.

In contrast to the acidic SVSPs, the basic forms have a glycine in lieu of the aspartate at S_1_ position 189 and this is the basis of their non-trypsin-like activity. They also have a glycine at positions 216 and 226, and consequently a large G189G216G226 substrate-binding pocket which would explain the preference for phenylalanine at P_1_ in the chromogenic substrates, but with a much lower specific activity than that of chymotrypsin (Table 1). The action of the basic SVSP on short peptides revealed more about its peptide bond specificity (Fig. 7, Fig. 10) pointing to a strong preference for large aliphatic residues tyrosine and phenylalanine at the P_1_ and site. In addition to this, it was able to cleave the L-C (though the latter is in the trioxidised form) in insulin B, and F-H and H-L bonds in the angiotensin peptides. Other SVSPs have been predicted to have this same G189G216G226 substrate pocket (Vaiyapuri et al., 2012), and in a small number of these, G189 is contained within a distinct arginine-rich sequence (RRRIG) on the N-terminal side of the substrate-binding site, as is the case for the *B. arietans* basic SVSPs (RRRRIG, Fig. 11). A BLAST search found just four such proteins with both of these structural features, all in African snakes: rhinocerases 4 and 5 of *B. gabonica rhinoceros* (Vaiyapuri et al., 2011), an *E. ocellatus* serine protease (submitted to GenBank: ADE45139.1) and a basic SVSP, alpha-fibrinogenase ML-AF, from *M. lebetina* (Siigur et al., 2003). Of these, only the latter has been isolated and fully characterised (Fig. 11 shows its substrate-binding site), and as well as having the same alpha-fibrinogen specificity as the puff adder basic SVSP, it also displays the same inactivity towards tyrosine-esters (Samel et al., 2002), but it was able to cleave insulin B at Y-L (Mahar et al., 1987). ML-AF also shows a preference for phenylalanine and, unlike the *B. arietans* basic SVSP, was able to cleave insulin B at both of the adjacent F-F-Y peptide bonds. This difference in activity may be a consequence of the slight difference in the arginine-rich sequence of the two; ML-AF has a proline in lieu of the first R in the RRRRIG of the puff adder protein (Fig. 11). Despite this small variation, it seems that ML-AF, described as unique in Siigur et al. (2003), and the *B. arietans* basic SVSP are related proteins. Alpha-fibrinogen degradation may be their principal role, contributing to an anticoagulant action of the respective venoms.

One conclusion of this work is that the *B. arietans* SVSPs are atypical biochemically, and it is difficult to place them within the traditional SVSP classification (Serrano and Maroun, 2005): trypsin-like acidic SVSPs which act against plasma proteins involved in haemostasis, and trypsin-like basic SVSPs with direct platelet-activating (PA) activities. The *B. arietans* acidic SVSPs appear to conform to this sub-classification (trypsin-like, fibrinogen degrading), but no previous studies have found gelatinase activity associated with this sub-class of SVSP. The basic SVSPs definitely do not fit within this classification, not least because they are not trypsin-like. In addition, they are structurally different from the known basic PA-SVSPs (e.g. PA-BJ (Serrano et al., 1995) and the thrombocytins (Niewiarowski et al., 1979; Hill-Eubanks et al., 1979)) which are lot smaller (28-29 kDa possessing just one N-glycan) than the puff adder basic SVSP.

The main purpose of this work was to expand our knowledge of the puff adder SVSPs which were identified to be a quantitatively significant component of the Nigerian venom in previous studies. Here, much has been revealed concerning their biochemistry, but to extrapolate the results of the assays to possible pathological effects can only be done at this stage by comparison with what is known from studies on other venom proteases. The ability of the gelatinolytic acidic SVSPs to degrade collagen I, the major form of collagen in mammalian tissues, suggests it may play a role in many of the clinical features of puff adder bites that might arise as a consequence of tissue damage. These would include damage at the bite site, often leading to necrosis, but also blisters which commonly form distal from the bite (Warrell et al., 1975; Tianyi et al., 2024). It has been proposed that snakebite-induced blistering can arise due to damage to fibrillar collagens at the dermal-epidermal junctions (Gutiérrez et al., 2018; Jiménez et al., 2008) and the gelatinolytic puff adder SVSPs may be responsible for this.

Both forms of SVSP cleaved fibrinogen in a manner typical of SVSPs. The acidic SVSPs cleaved both alpha and beta fibrinogen in a pattern that could be described as thrombin-like, as might be expected for a trypsin-like SVSP, whereas the basic SVSPs demonstrated alpha-only fibrinogenase activity. The combined activity of both forms of SVSP towards fibrinogen might contribute to the coagulopathy observed in puff adder envenomation by depleting functional fibrinogen and impairing its clottability by thrombin. Such a role for SVSPs is well documented (Serrano and Maroun, 2005; Kini, 2005), but in addition to this, the ability of the acidic forms to degrade collagen may also exacerbate the haemotoxic effects of the venom, since this protein plays a key role in the initial adherence of platelets to damaged blood vessel wall (Jackson, 2007).

We were able to study the possible role of these SVSPs in contributing to hypotension that is commonly seen in puff adder bite victims. The basic SVSP isolated from the Nigerian venom converted angiotensin I (1-10) to the vasoconstrictor angiotensin II (1-8) in contrast to the PI SVMPs present in all regional puff adders which convert it to the vasodilator angiotensin 1-7 (Wilkinson et al., 2026). Venoms of a type similar to the Nigerian used here, with both basic SVSPs and PI SVMPs, will thus produce two forms of angiotensin with contrasting actions, which we have shown experimentally (Table S4). It is difficult to predict the effect that this will produce upon envenomation, as the two forms of angiotensin may cancel each other out. For venoms of the Tanzanian type, with little SVSP but a potent PI SVMP, the prediction is more straightforward, with vasodilation and consequent hypotension being the most likely outcome as a consequence of its ability to generate only the angiotensin 1-7 peptide from its precursor.

Kallikrein-like SVSPs, with the ability to generate vasodilatory kinins, have been shown to play a role in inducing hypotension following a puff adder bite (Sekoguchi et al., 1986; Nikai et al., 1993 Megale et al., 2018) and such SVSPs are likely to be found amongst the acidic forms isolated here. The latter were active against the S-2302 chromogenic substrate (Table 1) that is used as specific substrate for kallikrein-like activity. They were also active against the equivalent tissue plasminogen activator (tPA) substrate, S-2288, but according to the manufacturers this can be used as a general substrate for serine proteases, and so this result does not suggest that these proteases have both tPA-like and kallikrein-like activities. To support the idea that the acidic SVSPs may be kallikrein-like, we have found that within the acidic SVSP transcripts are sequences that match tryptic peptide sequences (DIMLIR, TLCAGVLEGGK and HPCAQPHLPAFYTK) from the kallikrein-like SVSP, Kn-Ba, isolated by Megale et al. (2018). Thus, all 14 acidic SVSPs possess TLCAGVLEGGK, have DIMLIR or the closely related DIMLIK, and at least 10 residues matching the HPCAQPHLPAFYTK sequence. One of the NGA acidic SVSP transcripts (NIG_SVSP_7 Contig4601||Toxin2176, see Fig. 11) has a 100% match to all three KnBa tryptic peptides. Acidic SVSP7 is virtually identical in and around the substrate-binding site to the SVSPs 2 and 5 identified as the most likely forms of the acidic SVSPs isolated here and used in the chromogenic kallikrein substrate assays. It is likely, therefore, that all the acidic SVSPs in puff adder have kallikrein-like activity.

It would be difficult to determine the potential contribution of the SVSP-generated angiotensins and kinins to hypotension, as either could be one of many suspected causes of this clinical feature of puff adder envenoming. Hypotension can be an indirect result of venom-induced haemorrhage but could also arise from the direct action of other puff adder venom components known to cause vasodilation, such as adenosine (Graham et al., 2005), bradykinin potentiating peptides (Kodama et al., 2015) and vEGF-like proteins (Currier et., 2010).

## 5 Conclusion

This study has identified the presence of two atypical forms of SVSP in puff adders. One, an acidic form, is responsible for the strong gelatinase activity we previously detected whilst characterising puff adder SVMPs (Wilkinson et al., 2026). Usually associated with SVMPs, this gelatinase activity is a novel role for an SVSP. At least as important as this biochemical peculiarity is the observation that this acidic SVSP gelatinolytic activity is geographically variable, which will further compound the issues discussed in our prior SVMP paper (Wilkinson et al., 2026). In that study we identified two forms of an SVMP PI, one of which was much more destructive towards protein substrates, particularly ECM components, than the other. The presence of these two SVMPs in puff adder venoms also varied geographically. In the case of the two main venoms we have studied, the Nigerian venom has the weaker SVMP PI but has the gelatinolytic SVSP. In contrast the Tanzanian venoms had the more potent SVMP PI, which is particularly destructive towards laminins, but no gelatinolytic activity. Others, e.g. Ghana and Eswatini had both the potent SVMP PI and the gelatinase SVSP, and in the previous study (Wilkinson et al., 2026) the gelatinase activity in Kenyan venoms (with the weaker SVMP PI) was due to an SVMP PIII-c rather than an SVSP. Without a specific assay, it would be difficult to study any variation in the presence of the basic SVSP also identified here, but analysis of the transcripts suggests that the Tanzanian is particularly low in the level of the basic form of SVSP (Dawson et al., 2024). Since all of these proteolytic activities are likely to play a major role in snakebite pathology, it is not surprising, therefore, that the clinical features associated with puff adder envenomation are greatly variable from bite to bite. And it is evident from these two studies that development of a pan-African therapeutic for puff adder snakebite will need to accommodate this geographical variation in the levels of key venom components and their relative toxicity. Small molecule therapeutics (SMTs) are being considered as one way forward in the treatment of snakebite. SMT studies are generally aimed at SVMPs and phospholipases A_2_ in vipers, including puff adders (Albulescu et al., 2020). Whilst SVSPs have sometimes been targeted with the use of nafamostat, this acts only on trypsin-like serine proteases, so the question of whether the basic proteases in puff adder, and other venoms, play a role in snakebite pathology needs further elucidation.

## CRediT authorship contribution statement

**Mark C. Wilkinson:** Writing – original draft, Investigation, Conceptualization. **David T.K. Waterhouse:** Investigation. **Cassandra M. Modahl:** Writing – review & editing, Formal analysis. **Anthony J. Saviola:** Investigation. **Frank-Leonel Tianyi:** Writing – review & editing. **Robert A. Harrison:** Resources, Funding acquisition. **Nicholas R. Casewell:** Writing – review & editing, Resources, Funding acquisition.

## Declaration of competing interest

The authors declare that they have no known competing financial interests or personal relationships that could have appeared to influence the work reported in this paper.

## Data Availability

Data will be made available on request.
