# Supplementary material for: Structural and functional characterisation of isolated puff adder (Bitis arietans) venom serine proteases
Source: Toxicon X. 2026 Sep 1;32:100263. doi: 10.1016/j.toxcx.2026.100263 (PMC13599656; doi:10.1016/j.toxcx.2026.100263)
Supplement: Supplementary file 1 [file mmc1.docx]

**Supplementary figures** [Wilkinson et al 2026b]

**A**

**B**

**A**

**B**

75

75

50

50

**9	10	11**

**1	2	3	4	5	6	7	8	M**

**D**

**C**

75

50

70

50

**16	17**

**12	13	14	15**

**E**

75

50

**18	19	20	21	22	23**

**Fig. S1.** **Gelatinase activity (gelatin zymogram) of venoms taken from various African and Saudi Arabian puff adders.** The zymograms were prepared with gelatin included at 0.1% (Gel A, supplied by Thermo-Fisher) or 0.2% (Gels B-E, made in-house) in a 10% acrylamide SDS-PAGE gel. The gels were run under non-reducing conditions and 2 μg of venom was loaded per lane. The zymograms were then incubated as in section 2.4.5.1 and then visualised by staining with Coomassie Blue R250. **Gel A:** Venoms from snakes held at LSTM at the time of the study. Lanes 1 - 4: NGA and lanes 5-8: TZA. **Gel B:** Lanes 9 – 11, venoms from three captive bred Kenyan snakes (LSTM). **Gel C:** Lane 12, Venom from LSTM Eswatini snake; lane 13, stock Zimbabwe venom; lanes 14 and 15, stock South African venoms. **Gel D:** lanes 16, 17, venoms from two Ghanaian snakes formerly housed at LSTM. **Gel E:** Lanes 18 - 21 venoms from four Nigerian snakes formerly housed at LSTM; lane 22 Saudi Arabian stock venom; lane 23, Malawi Stock venom. The numbers to the right of each gel indicates the position of key molecular weight markers (kDa). These were run under reducing conditions and visualised following extensive de-staining of the gel.

Elution volume (mL)

Absorbance 280 nm (mAu)

6

5

7

4

3

2

1

25

50

35

75

100

10	11	12	13	15	16	17	18	19	M

**Fig. S2. Size exclusion chromatography separation of NGA *B. arietans* venom**. Whole venom (20 mg NGA 011) was separated on a 120 mL column of Superdex 200HR equilibrated in 50 mM sodium phosphate pH 5.2. The flowrate was 1.0 mL/min and elution was monitored at 280 nm. **A.** Chromatography trace, monitored at 280 nm [blue], with fractions shown in grey. **B.** SDS-PAGE of fractions from the first 3 peaks. The gel used was a 4-20% acrylamide (BioRad) and was stained with Coomassie Blue R250. Lane M, molecular weight markers (Promega Broad Range) with key molecular weights indicated in kDa.

**Table. S3. Peptide masses observed following digestion of insulin B chain with the purified basic SVSP.**

|  | m/z | z | m | peptide |
| --- | --- | --- | --- | --- |
|  | 416.25 | 1 | 415.25 | TPKA |
|  | 379.20 | 2 | 756.40 | FVNQHL |
|  | 1139.50 | 1 | 1138.50 | CGSHLVEALY |
|  | 1238.50 | 1 | 1237.50 | LVCGERGFFY |
|  | *619.77* | *2* | *1237.57* | *LVCGERGFFY* |

*Note: the insulin B chain used is oxidised with both cysteines as the trioxide form. This is taken into account [+48.00 Da] in the mass calculations*

| Protease or venom | Substrate | Products | | | | | |
| --- | --- | --- | --- | --- | --- | --- | --- |
|  |  | **m/z** | | **z** | **m** | **peptide** | |
|  |  |  | |  |  |  |  |
| Basic SVSP | Angiotensin I | *269.16* | | *1* | 268.16 | HL |  |
|  |  | 349.52 | | 3 | 1045.56 | DRVYIHPF | Ang 1-8 |
|  |  | 523.77 | | 2 | 1045.54 |  |  |
|  |  | 1046.51 | | 1 | 1045.51 |  |  |
|  |  | |  |  |  |  |  |
| Basic SVSP | Angiotensinogen 1-14 | | 395.20 | 3 | 1182.60 | DRVYIHPFH | Ang 1-9 |
|  |  | 366.21 | | 2 | 730.42 | HLLVYS |  |
|  |  | 523.77 | | 2 | 1045.54 | DRVYIHPF | Ang 1-8 |
|  |  | 432.90 | | 3 | 1295.70 | DRVYIHPFHL | Ang I (1-10) |
|  |  |  | |  |  |  |  |
| Tanzanian venom | Angiotensin I | 416.23 | | 1 | 415.23 | FHL |  |
|  |  | 450.24 | | 2 | 898.48 | DRVYIHP | Ang 1-7 |
|  |  |  | |  |  |  |  |
| Nigerian venom | Angiotensin I | 416.23 | | 1 | 415.23 | FHL |  |
|  |  | 450.24 | | 2 | 898.48 | DRVYIHP | Ang 1-7 |
|  |  | 523.77 | | 2 | 1045.54 | DRVYIHPF | Ang 1-8 |

**Table S4. Peptide masses observed following digestion of angiotensin peptides with purified basic SVSP or whole venoms**

*Angiotensinogen 1-14 = DRVYIHPFHLLVYS (m = 1757.93); Angiotensin I (1-10) = DRVYIHPFHL*
